# Does metformin really reduce prostate cancer risk: an up-to-date comprehensive genome-wide analysis

**DOI:** 10.1186/s13098-024-01397-7

**Published:** 2024-07-12

**Authors:** Xinxing Zhang, Zhen Li

**Affiliations:** 1 Chengdu New Radiomedicine Technology Co. Ltd, Chengdu, Sichuan China; 2 Department of Urology, Hunan Provincial People’s Hospital, The First Affiliated Hospital of Hunan Normal University, Changsha, Hunan China

**Keywords:** Metformin, Prostate cancer, Mendelian randomization, PRACTICAL, FinnGen

## Abstract

**Background:**

The relationship between metformin use and prostate cancer (PCa) risk has yet to be clear despite more than a decade of debate on this topic. Hence, we aimed to investigate the causal role of metformin in reducing PCa risk through an up-to-date comprehensive genome-wide analysis.

**Methods:**

We employed validated instrument variables of metformin use derived from a prior high-quality study, including five potential targets (AMPK, GCG, GDF15, MCI and MG3). Mendelian randomization (MR) analysis was performed to harmonize genetically predicted metformin use and PCa phenotypes. PCa phenotypes were from two large genome-wide association studies (GWAS), the Prostate Cancer Association Group to Investigate Cancer-Associated Alterations in the Genome (PRACTICAL) and the FinnGen cohort. Seven methods were applied to generate MR results: the inverse variance weighted (IVW), IVW with multiplicative random effects, MR-Egger, MR-Egger (bootstrap), weighted median, simple mode and weighted mode. Strict sensitivity analysis was conducted to satisfy core assumptions of MR design.

**Results:**

We enrolled 32 significant single nucleotide polymorphisms (SNPs) that involved with metformin use. Nearly all targets yielded insignificant primary results (IVW with multiplicative random effects), except that AMPK target posed a positive effect on PCa risk from FinnGen cohort [odds ratio (OR): 6.09, 95% confidence interval (CI): 1.10-33.53, P value: 0.038]. The general effect of metformin use, comprising all 5 targets, also yielded negative results (random-effect meta-analysis with OR: 1.09, 95% CI: 0.76–1.58, P value: 0.637 for PRACTICAL; OR: 2.55, 95% CI: 0.58–11.16, P value: 0.215 for FinnGen). None of the sensitivity analyses provided support for a causal association between metformin use and PCa risk.

**Conclusion:**

This up-to-date study did not support the protective role of metformin in reducing PCa risk, considering each target, overall effect, and sensitivity analysis. It is imperative to reflect on the presumed “almighty medicine” and ongoing phase III trials are anticipated to assess the anti-neoplasm effect of metformin.

**Supplementary Information:**

The online version contains supplementary material available at 10.1186/s13098-024-01397-7.

## Introduction

Metformin is a widely used pharmacological agent for the management of type 2 diabetes mellitus, which has attracted growing attention due to its potential anti-tumorigenic characters [1]. Although the contentious conclusions have left this matter unresolved, the efficacy of metformin in diverse cancer types has been elucidated in an escalating number of clinical investigations [2–14].

The association between metformin use and prostate cancer (PCa) risk is of great interest to urologists. Previous studies indicated the protective effect of metformin in at least a certain part of population [8, 15–23], while some others contradicted the results [24–33]. As observational studies are rife with numerous disadvantages [34], a genetic tool is appropriate to investigate the causal association between metformin and PCa. Mendelian randomization (MR) analysis is an effective method to solve such issues [35–37]. But the frustrating reality is that only one study focused on this topic [38]. The study utilized the target of adenosine 5’-monophosphate-activated protein kinase (AMPK) to proxy metformin effect on HbA1c reduction, which was inaccurate and biased due to its complicated effect [38].

Recently, a comprehensive research summarized the distinct drug target impacts of metformin through genome-wide analysis [39]. As a result, we are able to exploit such instruments to explore the effect of metformin on PCa, which is the aim of this study.

## Methods

**Fig. 1:**
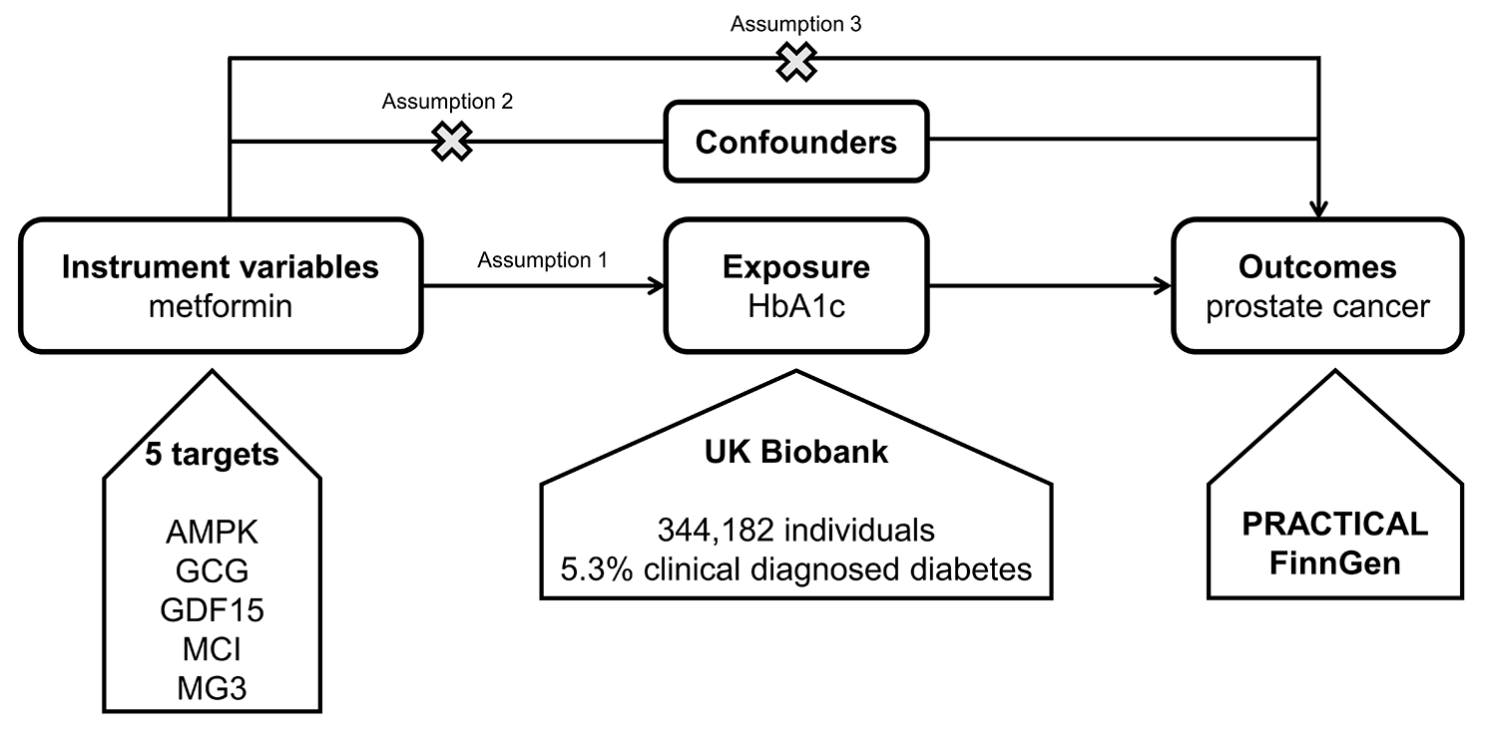
The study flowchart. AMPK: adenosine 5’-monophosphate-activated protein kinase; GCG: glucagon; GDF15: growth differentiation factor 15; MCI: mitochondrial complex I; MG3: mitochondrial glycerol 3; PRACTICAL: Prostate Cancer Association Group to Investigate Cancer-Associated Alterations in the Genome

The objective of this study is to test the causal effect of metformin use on PCa risk through a comprehensive MR analysis. Figure 1 showed the study flowchart.

### Metformin proxied instrument variables

We utilized the certified variants of metformin use from a previous high-quality study [39]. Briefly, the authors determined 5 targets (AMPK, GCG, GDF15, MCI, MG3) with 32 variants through a series of validation. They conducted a thorough literature review to identify the drug targets of metformin (AMPK, GCG, GDF15, MCI, MG3). The five metformin-related targets were then mapped to the related genes through the ChEMBL database [40, 41]. Furthermore, the related genes were mapped to the related genetic variants based on recent comprehensive data [42–48]. The related genetic variants were then associated with the glycemic trait HbA1c from 344,182 UK Biobank participants (served as the exposure variable, Fig. 1) and we provided the summary-level data in Table S1.

### Prostate cancer outcomes

We selected PCa outcomes from the Prostate Cancer Association Group to Investigate Cancer-Associated Alterations in the Genome (PRACTICAL) and the FinnGen cohort. PRACTICAL is a consortium to investigate the genetic susceptibility of PCa, consisting of 79,148 cases and 61,106 controls [49]. As for FinnGen cohort, we extracted release 5 version data, consisting of 6,311 cases and 88,902 controls [50]. Only European ancestry was included and overlap was avoided between the exposure and outcome variables. Details were provided in Table S2.

### Statistical analysis

All the analysis was completed in R (version 4.2.0). TwoSampleMR and ieugwasr were the main R packages. Two-sample MR analysis was the primary results in our study. F-statistic, calculated as beta^2^/se^2^, was implemented to test the power of instrument variables, with F-statistic > 10 thought as strong variants [51]. When conducting MR analysis, proxy with r^2^ > 0.8 was considered if a single nucleotide polymorphism (SNP) was not matched between the exposure and outcome variables. We enrolled seven methods to generate MR results: the inverse variance weighted (IVW), IVW with multiplicative random effects, MR-Egger, MR-Egger (bootstrap), weighted median, simple mode and weighted mode. The IVW with multiplicative random effects method was considered as our primary result. If there was only one SNP in the exposure and outcome variables, Wald ratio was calculated as the primary result. We would report MR results based on each target mentioned above and then give the whole results of the five targets. Additionally, heterogeneity and pleiotropy tests were conducted. All results were reported as odds ratio (OR) or beta value with 95% confidence interval (95% CI). On the other hand, Steiger tests were conducted to certify whether the assumption that exposure caused outcome was valid. A reverse MR analysis was also performed to examine if the reverse causality existed.

Three assumptions should be met during MR analysis (Fig. 1). First, all SNPs were associated with the exposure variable. Second, any SNP associated with any potential confounder should be excluded. Third, SNPs should not be associated with the outcome variable directly. To satisfy all these assumptions, we intended to perform the following sensitivity analysis. Sensitivity analysis 1 removed SNPs with F-statistic < 10. Sensitivity analysis 2 removed SNPs associated with hypertension additionally. Sensitivity analysis 3 removed SNPs associated with hypertension and dyslipidemia additionally. Sensitivity analysis 4 removed SNPs associated with hypertension, dyslipidemia and body mass index (BMI) additionally. Sensitivity analysis 5 removed SNPs associated with hypertension, dyslipidemia, BMI and any cancer outcome additionally.

## Results

We enrolled 32 significant SNPs from the previous research [39], including five targets (AMPK, GCG, GDF15, MCI, MG3) that involved with metformin use. Those 32 SNPs were associated with the genome-wide association study (GWAS) of a glycemic marker, HbA1c, from UK Biobank (18,242 diabetic cases/325,940 controls). To prevent overlap bias between the exposure and outcome variables that could induce false positive rate, we selected prostate cancer GWAS from another two UK Biobank-unrelated cohorts, PRACTICAL (79,148 cases/61,106 controls) and FinnGen (6,331 cases/88,902 controls), to perform MR analysis (Fig. 1).

### The MR effect of metformin targets on PCa risk

**Fig. 2:**
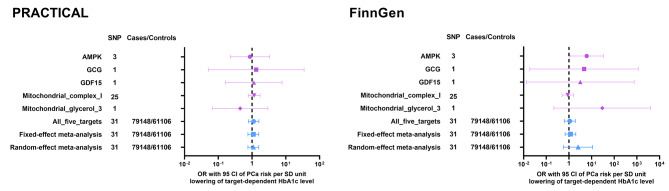
The MR effect of metformin use on PCa risk from PRACTICAL and FinnGen, based on each target and general effect. MR: Mendelian randomization; PCa: prostate cancer risk; PRACTICAL: Prostate Cancer Association Group to Investigate Cancer-Associated Alterations in the Genome; SNP: single nucleotide polymorphism; OR: odds ratio; CI: confidence interval; SD: standard deviation

We conducted the MR analysis of metformin use effect on PCa cancer risk based on each target (Fig. 2).Nearly all targets yielded insignificant primary results (IVW with multiplicative random effects), except that AMPK target posed a positive effect on PCa risk from FinnGen cohort (OR: 6.09, 95% CI: 1.10-33.53, P value: 0.038, Table S3B). The effect of AMPK target on PCa risk from PRACTICAL was of no significance (OR: 0.87, 95% CI: 0.23–3.33, P value: 0.835, Table S3A). The other four targets, including GCG (OR: 1.33, 95% CI: 0.05–34.81, P value: 0.865 for PRACTICAL; OR: 4.67, 95% CI: 0.02-1176.74, P value: 0.585 for FinnGen), GDF15 (OR: 1.13, 95% CI: 0.16–7.78, P value: 0.901 for PRACTICAL; OR: 3.17, 95% CI: 0.01-756.47, P value: 0.680 for FinnGen), MCI (OR: 1.16, 95% CI: 0.77–1.74, P value: 0.477 for PRACTICAL; OR: 0.89, 95% CI: 0.50–1.60, P value: 0.699 for FinnGen) and MG3 (OR: 0.45, 95% CI: 0.07–2.95, P value: 0.402 for PRACTICAL; OR: 29.76, 95% CI: 0.22-4085.54, P value: 0.177 for FinnGen), all showed negative results (Fig. 2, Table S3A and Table S3B).

The general effect of metformin use, comprising all 5 targets, also yielded negative results when we utilized methods of IVW with multiplicative random effects (OR: 1.12, 95% CI: 0.78–1.60, P value: 0.542 for PRACTICAL; OR: 1.09, 95% CI: 0.62–1.91, P value: 0.760 for FinnGen), fixed-effect meta-analysis (OR: 1.09, 95% CI: 0.76–1.58, P value: 0.637 for PRACTICAL; OR: 1.16, 95% CI: 0.68–2.01, P value: 0.584 for FinnGen) and random-effect meta-analysis (OR: 1.09, 95% CI: 0.76–1.58, P value: 0.637 for PRACTICAL; OR: 2.55, 95% CI: 0.58–11.16, P value: 0.215 for FinnGen; Fig. 2; Table 1, Table S3A and Table S3B). Steiger analysis showed correct causal direction from metformin use to PCa risk (Table S4A and Table S4B). In addition, the reverse MR analysis to examine if the reverse causality existed indicated no causal association between PCa and metformin use (P value of IVW with multiplicative random effects: 0.383 for PRACTICAL and 0.779 for FinnGen, Table S5A and Table S5B).

**Table 1:** The general effect of metformin use on prostate cancer risk

Exposure	Target	Outcome	SNP	Method	OR	LCI_OR	UCI_OR	Pval
HbA1c	All_five_targets	Prostate cancer (PRACTICAL)	31	Inverse variance weighted	1.117343843	0.782122926	1.596241744	0.5420757
				Inverse variance weighted (multiplicative random effects)	1.117343843	0.782122926	1.596241744	0.5420757
				MR Egger	0.65899054	0.327302272	1.326811846	0.2523115
				MR Egger (bootstrap)	0.815780953	0.528775051	1.258566496	0.1830000
				Weighted median	0.761102225	0.513982634	1.127035349	0.1729054
				Simple mode	0.855173933	0.423197232	1.728088942	0.6660282
				Weighted mode	0.763888686	0.524522126	1.11249058	0.1705234
				Fixed-effect meta-analysis	1.093080656	0.755028335	1.582490703	0.6368
				Random-effect meta-analysis	1.093080656	0.755028335	1.582490703	0.6368
HbA1c	All_five_targets	Prostate cancer (FinnGen)	31	Inverse variance weighted	1.090991505	0.588571936	2.02228885	0.7821014
				Inverse variance weighted (multiplicative random effects)	1.090991505	0.624023547	1.90739992	0.7599560
				MR Egger	0.595829742	0.17958971	1.976800791	0.4043435
				MR Egger (bootstrap)	0.598592167	0.234780011	1.526163067	0.1410000
				Weighted median	0.737403756	0.303724106	1.790323153	0.5008806
				Simple mode	0.915505818	0.183291609	4.572772907	0.9150478
				Weighted mode	0.694637925	0.290906462	1.658683839	0.4184030
				Fixed-effect meta-analysis	1.164160236	0.675028748	2.007720502	0.5844
				Random-effect meta-analysis	2.547213458	0.581002627	11.15625135	0.2151

SNP: single nucleotide polymorphism; PRACTICAL: Prostate Cancer Association Group to Investigate Cancer-Associated Alterations in the Genome; MR: Mendelian randomization

### Sensitivity analysis

To meet the three core assumptions of MR analysis, we performed five sensitivity analysis mentioned in the method section. There were 23, 22, 18, 16 and 16 SNPs enrolled in sensitivity analysis 1–5 respectively. The detailed SNPs information was provided in Table S6A-E.

**Fig. 3:**
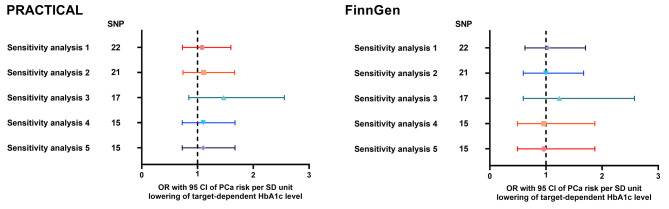
The MR effect of metformin use on PCa risk from PRACTICAL and FinnGen, based on sensitivity analysis. MR: Mendelian randomization; PCa: prostate cancer risk; PRACTICAL: Prostate Cancer Association Group to Investigate Cancer-Associated Alterations in the Genome; SNP: single nucleotide polymorphism; OR: odds ratio; CI: confidence interval; SD: standard deviation

All the sensitivity analysis did not support a causal association between metformin use and PCa risk. The effect sizes based on the IVW with multiplicative random effects were: sensitivity analysis 1 (OR: 1.08, 95% CI: 0.73–1.60, P value: 0.698 for PRACTICAL; OR: 1.03, 95% CI: 0.63–1.70, P value: 0.901 for FinnGen), sensitivity analysis 2 (OR: 1.11, 95% CI: 0.74–1.67, P value: 0.614 for PRACTICAL; OR: 1.00, 95% CI: 0.59–1.67, P value: 0.991 for FinnGen), sensitivity analysis 3 (OR: 1.47, 95% CI: 0.84–2.56, P value: 0.174 for PRACTICAL; OR: 1.24, 95% CI: 0.60–2.58, P value: 0.567 for FinnGen), sensitivity analysis 4 (OR: 1.10, 95% CI: 0.73–1.67, P value: 0.649 for PRACTICAL; OR: 0.96, 95% CI: 0.49–1.87, P value: 0.905 for FinnGen) and sensitivity analysis 5 OR: 1.10, 95% CI: 0.73–1.67, P value: 0.649 for PRACTICAL; OR: 0.96, 95% CI: 0.49–1.87, P value: 0.905 for FinnGen; Fig. 3, Table S7A and Table S7B).

## Discussion

In this study, we confirmed that no protective effect of metformin use on PCa risk. The association between metformin use and PCa risk reduction has been debated for over fifteen years [52, 53]. Most results were derived from in vitro or observational studies, as randomized controlled trials were impractical and the follow-up time was too long to gain enough events. Contradictory results were expected due to potential known or unknown confounders. Therefore, we conducted such a genetic epidemiological study to examine the hypothesis that whether metformin use causally reduced PCa risk, which had the advantage of test causality if all assumptions were satisfied. Unfortunately, we were unable to validate the preventive effect of metformin on PCa risk based on MR analysis of each target, all five targets and all the sensitivity analysis. Maybe we should re-examine the relationship between metformin use and PCa risk.

An early study has explored the impact of genetic-predicted metformin use on PCa risk [38]. However, the research just utilized the AMPK-proxied HbA1c reduction as a substitute of metformin, which was obsolete and far from comprehensive, as metformin exerted its effect not only through AMPK pathway activation. Also, the way it included SNPs in the MR analysis was with inferior priority to the recent study [39]. Au Yeung and colleagues found no causal association between metformin use and PCa risk in their conclusion [38], which was similar with our results. From this point of view, the utilization of metformin for PCa prevention should be cautious, at least metformin might not reduce PCa risk in a blood sugar dependent way.

Interestingly, one randomized controlled trial regarding the protective effect of metformin on anthropometric and metabolic complications in patients receiving radical radiotherapy and androgen deprivation therapy was completed and reported its preliminary results [20]. This phase II trial discovered that metformin did not attenuate the complication rate, which was frustrating. Nevertheless, metformin is currently under investigation in the further phase 3 trial to evaluate its potential anti-tumor effects. As far as we know, this is the first randomized controlled trial to investigate only the effect of metformin in prostate disease, although the aim is to evaluate its preventive impact in decreasing complication rate. But it did provide some information. Maybe metformin actually does not have the potency as we expected in antagonizing PCa. We ought to be vigilant when considering the effect of such an “almighty medicine”. We are also looking forward to the further results of the phase 3 trial [20].

The study tried to solve the long discussed issue. We utilized the design of MR to avoid confounder bias and and intended to establish a causal association. We incorporated an up-to-dated comprehensive genetic proxy of metformin into our study to explore its role in PCa risk. Apart from the above advantages, we divided the metformin effect into several targets and calculated the specific effect of each target. Nearly all targets yielded no significant results, which indeed confirmed no causal relationship between metformin use and PCa risk. Moreover, all the three key assumptions of MR analysis were met and we conducted several sensitivity analysis to validate our results. We believe our research could offer information to those urologists who are interested in medical treatment of PCa.

Some limitations should be admitted. First, we just enrolled European ancestry in this study resulting from a lack of summary statistics from other ancestries. Additionally, there might be some other targets through which metformin functioned, but we have not discovered till now. Notwithstanding, we summarized the current evidence of metformin effect on PCa. The results might alter as further targets of metformin are found.

To conclude, the study did not find a reliable causality between metformin use and PCa risk, based on each target, general effect or sensitivity analysis. We should reflect on the “almighty medicine” and doubt its protective effect of PCa risk. Perhaps metformin influences PCa through other rather than glycemic pathway. The ongoing phase III trial is anticipated as it would assess the anti-neoplasm effect.

### Electronic supplementary material

Below is the link to the electronic supplementary material.

Supplementary Material 1

## Data Availability

All data generated or analyzed during this study are included in this published article and its supplementary information files.
